# Serum IgM Glycosylation Associated with Tuberculosis Infection in Mice

**DOI:** 10.1128/mSphere.00684-18

**Published:** 2019-03-27

**Authors:** Tadahiro Kumagai, Ainhoa Palacios, Arturo Casadevall, M. Jesús García, Carlos Toro, Michael Tiemeyer, Rafael Prados-Rosales

**Affiliations:** a CIC bioGUNE, Derio, Bizkaia, Spain; b Complex Carbohydrate Research Center, The University of Georgia, Athens, Georgia, USA; c Department of Molecular Microbiology and Immunology, Johns Hopkins Bloomberg School of Public Health, Baltimore, Maryland, USA; d Department of Preventive Medicine, Public Health and Microbiology, Autonomous University of Madrid, Madrid, Spain; e Service of Microbiology, Hospital Universitario La Paz, IdiPaz, Madrid, Spain; f Department of Microbiology and Immunology, Albert Einstein College of Medicine, Bronx, New York, USA; University of Maryland School of Medicine

**Keywords:** IgM, *Mycobacterium tuberculosis*, fucosylation, glycans, immunoglobulin M, mice

## Abstract

Changes in serum glycans discriminate between disease statuses in cancer. A similar connection has not been established in the context of infectious diseases such as tuberculosis (TB). The inflammation arising from infection by Mycobacterium tuberculosis may affect host protein glycosylation, thereby providing information about disease status in TB. A mouse model of infection was used to study glycoprotein N-glycosylation in serum. Following digestion of serum glycoproteins with peptide-*N*-glycosidase F (PNGase F), released glycans were permethylated and analyzed by multidimensional mass spectrometry (MS). Conditions included naive or Mycobacterium bovis BCG-vaccinated animals, which were either uninfected or infected with *M. tuberculosis*. MS results were validated by lectin blotting. We found that both glycoprotein fucosylation and sialylation were particularly sensitive to *M. tuberculosis* infection. We observed that *M. tuberculosis* infection elevates serum IgM levels and induces changes in glycosylation that could inform about the disease.

**IMPORTANCE** We demonstrate that *M. tuberculosis* infection influenced host protein glycosylation in a mouse model. The mechanism by which infection modifies glycans in serum proteins is not understood. Investigation of the regulation of such modifications by *M. tuberculosis* opens a new field that could lead to the discovery of novel biomarkers. Validation of such findings in human samples will reveal the clinical relevance of these findings.

## Abstract (precis)

We demonstrate that *M. tuberculosis* infection influenced host protein glycosylation in a mouse model. The mechanism by which infection modifies glycans in serum proteins is not understood. Investigation of the regulation of such modifications by *M. tuberculosis* opens a new field that could lead to the discovery of novel biomarkers. Validation of such findings in human samples will reveal the clinical relevance of these findings.

## INTRODUCTION

The glycome represents the entirety of carbohydrates present in molecules, cells, and tissues. A significant body of data exists indicating that changes in the glycome are linked to disease and, conversely, that infection and inflammation lead to changes in the glycome (1). Considering that the host glycome is generated by a number of enzymes, including glycosyltransferases, glycosidases, and nucleotide sugar transporters acting at the level of the Golgi complex, any change in the metabolic status of the cell can lead to changes in the sugar moieties added to proteins. Consequently, infection by a pathogen and the associated inflammatory response may affect host glycosylation. Interestingly, aberrantly glycosylated proteins are a hallmark of oncogenic transformation, providing useful biomarkers for cancer progression (2). Moreover, recent advances in glycoanalytical methods have facilitated high-throughput screens for these serum biomarkers (3). Although serum glycome analysis seems to be a reliable method to discriminate between cancer and control patients, there is little or no information supporting the notion that glycome changes correlate with states of infection by a specific pathogen or with protection by vaccination. Recently, a correlation between tuberculosis (TB) disease status in humans and glycosylation changes in immunoglobulin Gs (IgGs) was demonstrated (4). IgGs isolated from individuals with latent TB possessed enhanced Fc effector functions and drove macrophages to kill intracellular Mycobacterium tuberculosis, indicating that differential glycosylation of IgGs, not just differential expression of IgG subclass, can modulate the capacity to control bacterial replication. In fact, this dimension of IgGs has already been extensively studied in the context of other infectious agents, including influenza virus (5), HIV (6–8), Ebola virus (9), and malaria agents (10).

The host response to *M. tuberculosis* plays a major role in the determination of the different clinical manifestations of the disease. *M. tuberculosis* can persist within the host for a lifetime, where it can reactivate due to various stimuli, including HIV infection, diabetes, cancer, or malnutrition (11). In 2015, the WHO reported an estimated 9.4 million new cases of TB, with an estimated 1.5 million deaths (12). Despite the fact that there are tools already in place to control the disease, TB is still the leading cause of mortality and morbidity by a curable bacterial infection in the developing world. The inflammation generated upon *M. tuberculosis* infection is necessary for the host defense, yet can result in immunopathologic damage (13). It is assumed that a balanced immune response that includes both pro- and anti-inflammatory responses is essential for controlling bacterial proliferation within granulomas. Considering the magnitude and diversity of the anti-TB immune response, we hypothesized that serum glycome changes may occur and that these changes may inform about the disease status of the host.

In this study, we study broad changes in serum glycoproteins beyond IgGs from naive, infected, and Mycobacterium bovis BCG-vaccinated murine models of infection.

## RESULTS

### Protective efficacy of BCG vaccination in murine model of *M. tuberculosis* infection.

**FIG 1:**
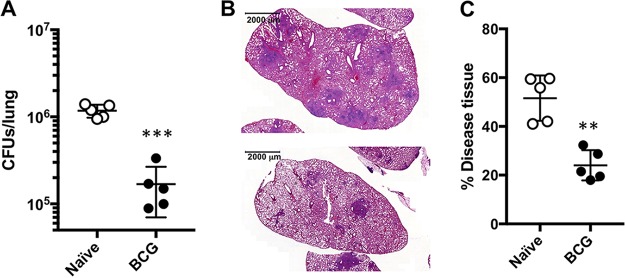
Protective efficacy of BCG immunization against M. tuberculosis infection. (A) CFU in the lungs of individual C57BL/6 mice, immunized with 10^6^ BCG bacteria 4 weeks after infection with a low dose of *M. tuberculosis* H37Rv via aerosol (approximately 100 CFU). The results are pooled values from two similar and independent experiments. (B) Representative H&E staining images from lungs of C57BL/6 mice sham immunized (top) and BCG immunized (bottom) and aerosol infected with *M. tuberculosis* H37Rv for 4 weeks. (C) Morphometric analysis of lung histopathology by assessing the percentage of diseased tissue. Experimental groups used 5 mice (**, *P < *0.01; ***, *P < *0.001).

We reproduced a typical BCG vaccination experiment where mice were immunized with live BCG prior to an infection with the virulent strain of *M. tuberculosis*. We observed a significant 1 log of reduction in the number of lung CFU in vaccinated versus unvaccinated mice 4 weeks after infection (Fig. 1A). This enhanced control of bacterial replication in the lung correlated with decreased lung pathology, as shown by reduced areas of inflammation measured as the percentage of diseased tissue (Fig. 1B and C). We hypothesized that N-glycan changes must be occurring during *M. tuberculosis* infection and that the beneficial effect of BCG vaccination must also impact the N-glycan structures of serum glycoproteins. To do this, we isolated serum from the same animals in Fig. 1.

### Alteration of the serum glycome upon *M. tuberculosis* infection.

**FIG 2:**
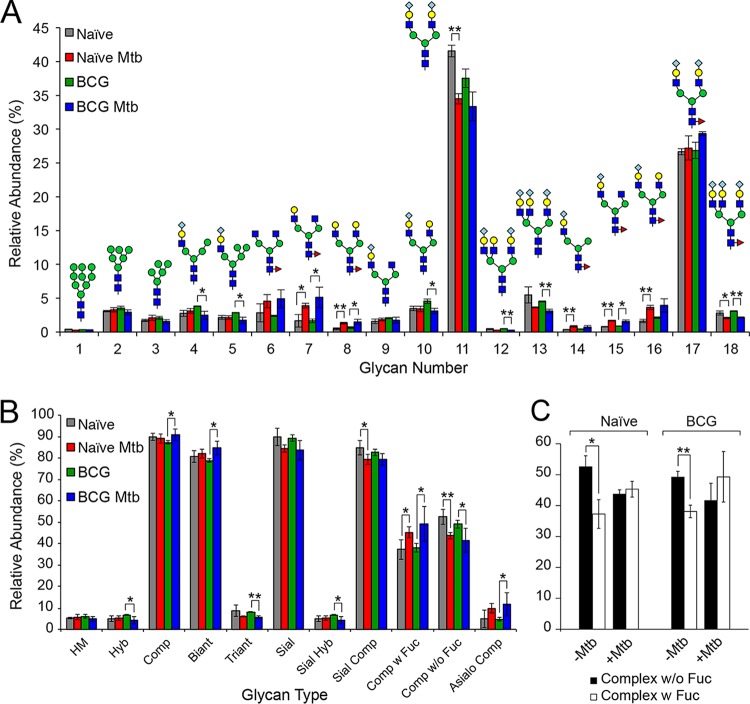
Serum N-glycans from mouse models of *M. tuberculosis* infection. (A) Based on the intensities of N-glycan signals for each structure, the prevalence of each N-glycan was quantified as a percentage of the total N-glycan intensity in the indicated serum samples. The relative abundance calculated for each glycan is plotted as the average from three independent measurements (biological replicates) with standard errors. Gray, red, green, and blue bars indicate naive control, naive following *M. tuberculosis* infection, BCG control, and BCG following *M. tuberculosis* infection, respectively. (B) To compare the relative abundance of each type of glycan across disease status, the total relative abundance of each glycan type was summed separately for each group. (C) To compare the relative abundance of core fucosylation across disease status, the total relative abundance of fucosylated and nonfucosylated glycans was summed separately for both naive and BCG-vaccinated mice. Black bars and white bars indicate nonfucosylated complex-type N-glycan and core fucosylated N-glycan, respectively. The averages from three independent experiments with standard errors are shown. (*, *P < *0.05; **, *P < *0.01; both panel A and panel B).

We investigated glycoprotein N-glycosylation in serum of mice infected with *M. tuberculosis* by comparing the N-glycans released from serum proteins harvested from naive and BCG-vaccinated mice with their *M. tuberculosis*-infected counterparts (Fig. 2 and data not shown). Full mass spectrometry (MS) analysis (data not shown) detected the expression of high-mannose-type, hybrid-type, complex-type biantennary, and complex-type triantennary glycans (Fig. 2). The major N-glycans were disialylated, biantennary complex glycans with or without core fucosylation (structures 11 and 17 in Fig. 2).

Comparing the serum glycomes of infected and naive mice, with and without BCG vaccination, changes in glycosylation patterns were detected. The disialylated biantennary complex glycan (structure 11 in Fig. 2A) was significantly decreased in unvaccinated *M. tuberculosis*-infected mice relative to unvaccinated naive mice. Significant decreases were observed in di- and trisialylated triantennary complex glycans (structures 13 and 18 in Fig. 2A) in BCG-vaccinated and infected mice relative to vaccinated and uninfected mice. Conversely, several core fucosylated complex glycans were significantly increased in *M. tuberculosis*-infected mouse serum (structures 14, 15, and 16 in Fig. 2A). When assessing differences in the relative abundance of glycan types, we observed a significant increase in complex and biantennary glycans in samples from immunized and infected mice relative to mice only immunized (Fig. 2B). We observed the opposite trend in triantennary and sialylated and hybrid-type glycans. No change was generally observed between naive and *M. tuberculosis*-infected mice except for the significant reduction in *M. tuberculosis*-infected mice in the relative abundance of sialylated complex-type glycans (Fig. 2B). The general theme of decreased nonfucosylated complex and increased fucosylated complex glycans in *M. tuberculosis*-infected mice was broadly consistent when comparing naive and BCG-immunized mice. To assess the relevance of altered fucosylation for infection and vaccination responses, we quantified the relative abundance of all N-glycans with or without core fucose (Fig. 2C). In naive and BCG-vaccinated/uninfected mice, nonfucosylated glycans were present in significantly higher abundance than fucosylated glycans. However, infection with *M. tuberculosis* in naive or vaccinated mice resulted in serum glycan profiles in which fucosylated and nonfucosylated glycans were detected in similar abundance, consistent with increased glycan core fucosylation in response to *M. tuberculosis* infection, regardless of vaccination status.

**FIG 3:**
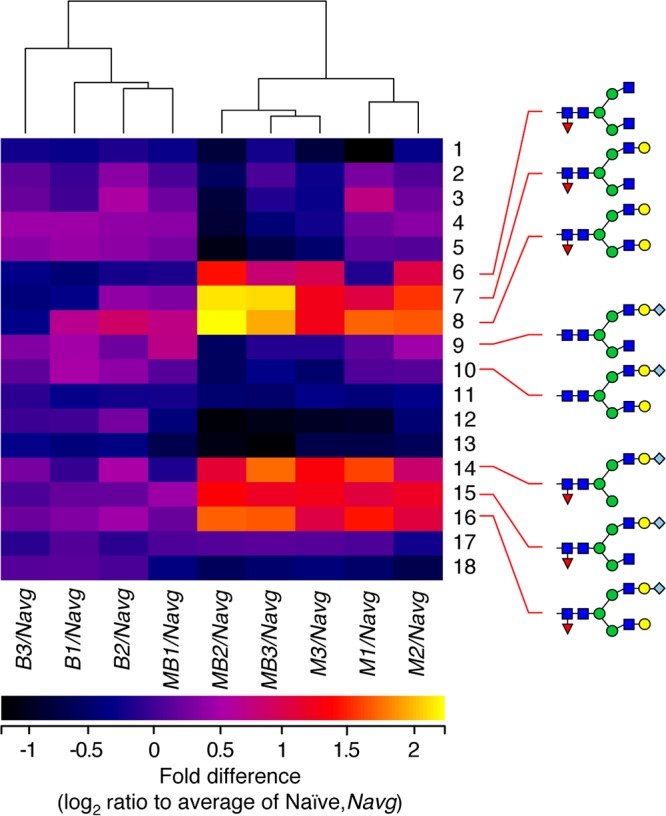
Hierarchical clustering of N-glycan relative abundances segregates mouse samples by disease state. N-glycans harvested from naive (*N*), *M. tuberculosis*-infected (*M*), BCG-vaccinated uninfected (*B*), or BCG-vaccinated and infected (*MB*) mice were quantified in three separate animals as biological replicates (designated 1 to 3 for each mouse type). Relative glycan abundance was calculated for each glycan as percentage of the total profile, and the values for each biological replicate were compared to the average of the abundance value for the naive mice (*Navg*). This ratio was defined as the fold difference in relative glycan abundance for each treatment condition relative to naive mice. The log_2_ of the ratio was taken to facilitate comparison across samples and glycan abundance scales such that a value of 0 corresponds to no change and each log increment corresponds to a 2-fold increase or decrease. Heat map clustering of the fold differences indicates that the N-glycan profile of BCG-vaccinated mice is similar to naive mice (*B/Navg*); all of the *B/Navg* replicates cluster together. However, five of the six biological replicates of infected mice, regardless of their vaccination status (*M* and *MB* replicates), exhibited glycan profiles distinct from the naive (*Navg*) and vaccinated uninfected (*B*) profile. The segregation of disease status was driven most forcefully by increased abundance of core fucosylated glycans (glycans 6 to 8 and 14 to 16).

To assess whether glycan profiles are predictive of disease or vaccination status, we performed hierarchical clustering using the relative abundance of each of the 18 glycans we quantified across the four sample types. We calculated the fold change in the abundance for each biological replicate of each sample type relative to the average value of the abundance for each glycan in the naive sample (Fig. 3). Using this fold change parameter, immunized mice exhibited glycomic profiles very similar to those of naive mice. However, clustering of the fold changes in the glycomic profile of *M. tuberculosis*-infected mice again highlighted increased core fucosylation as a major driver of the infection process, whether mice were immunized or not. The glycomic profiles of all but one of the infected mice clustered separately from the immunized and uninfected mice.

### Identification of serum glycoproteins with altered glycosylation upon *M. tuberculosis* infection.

**FIG 4:**
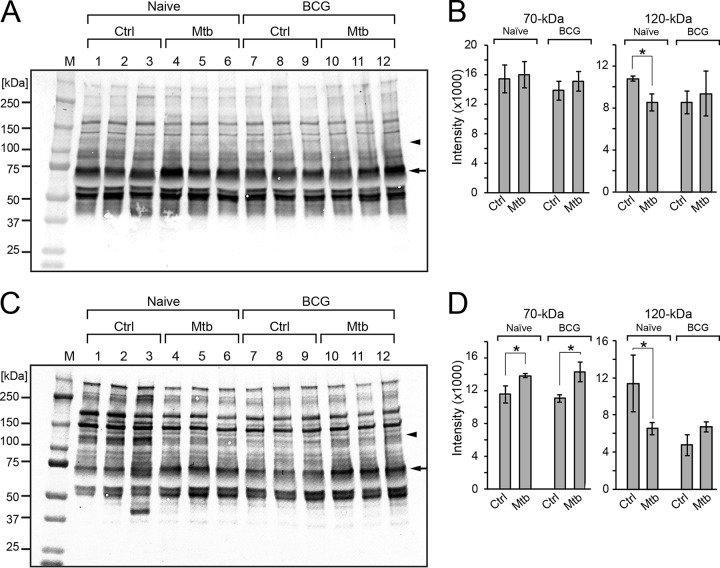
Detection of glycosylation differences by lectin blotting of serum proteins in infected mice. (A and C) Blotted mouse serum proteins were probed with AAL (A) and SNA (C). Naïve and BCG denote serum harvested from mice without or with BCG vaccination, respectively. Ctrl and Mtb denote serum harvested from mice without or with *M. tuberculosis* challenge, respectively. Lanes designated 1 to 12 indicate replicate serum samples. Intensities of the bands at 70 kDa and 120 kDa were quantified using ImageJ (B and D). The asterisk indicates *P < *0.05. The arrow and arrowhead indicate bands at 70 kDa and 120 kDa, respectively. M, molecular weight markers.

To identify glycoproteins with altered glycosylation, serum proteins were separated by SDS-PAGE, blotted onto PVDF membranes, and probed with lectins with well-defined binding specificities. Aleuria aurantia lectin (AAL) binds to N-glycans that are core fucosylated (Fig. 4A) (14, 15). AAL demonstrated a significant decrease in staining around 120 kDa in serum samples from *M. tuberculosis*-infected and unvaccinated mice (Fig. 4A and B). Sambucus nigra agglutinin (SNA) lectin also exhibited increased staining in this mass region of the blot, indicating enhanced terminal α2-6-linked sialylation (Fig. 4C and D) (16). SNA binding was also increased in the apparent mass range of 70 kDa in serum from *M. tuberculosis*-infected mice independent of their vaccination status (Fig. 4C and D). A significant increase in the binding of AAL to the 70-kDa gel region was not detected (Fig. 4A and B). These results suggest an association between *M. tuberculosis* infection and glycosylation changes in subsets of serum proteins.

In-gel tryptic digestion and subsequent liquid chromatography-tandem MS analysis (LC-MS/MS) indicated that the 70-kDa and the 120-kDa mass ranges are enriched in Igμ chain C region and the inter-alpha-trypsin inhibitor heavy chain (ITIH4), respectively (Table 1). LC-MS/MS proteomic identifications were validated by immunoblotting using specific antibodies to each protein (data not shown). Probing of immunoblots with anti-Igμ chain detected increased Igμ chain in *M. tuberculosis*-infected serum sample relative to serum from naive mice (data not shown), suggesting that the increase of binding to SNA could be due to an increase in the protein levels of Igμ chain. Interestingly, no changes in Igμ chain protein levels were observed in BCG samples, indicating that the increase in SNA binding reflects increased moles of sialic acid per mole of protein upon immunization. Validation of the ITIH4 protein assignment with N-terminal specific antibody revealed differential processing in serum from BCG-immunized/uninfected control relative to BCG-immunized/infected mice (data not shown). Considering that ITIH4 abundance changes have also been associated with other infectious (17) and noninfectious conditions, such as hepatocellular carcinoma (18) and ischemic stroke (19), the TB specificity of ITIH4 status was not investigated further, and we chose to focus our analysis on the glycosylation status of serum IgM.

**TABLE 1:** Protein identification in mouse serum samples by LC-MS/MS

Mass area (kDa)^*a*^	Identified protein	Score^*b*^	Coverage (%)	Mol wt (kDa)	Accession no.^*c*^
120	Inter-alpha-trypsin inhibitor heavy chain H4	20.63	1.08	102.8	E9Q5L2
7	Immunoglobulin Mu chain	75.34	27.47	50.0	A0A075B5P6
	Carboxylesterase 1C	45.71	20.58	61.0	P23953
	Hemopexin	18.44	16.96	51.3	Q91X72
	Histidine-rich glycoprotein	9.63	8.00	59.1	Q9ESB3
	Tetratricopeptide repeat protein 29	8.34	3.18	54.3	E9QLU4
	Vitronectin	7.20	5.44	54.8	P29788

a Molecular weight range for excised bands as indicated in Fig. 4.

b Probability score derived from Proteome Discoverer 1.4 analysis.

c UniProt accession number.

### BCG vaccination impacts glycosylation of IgM.

**FIG 5:**
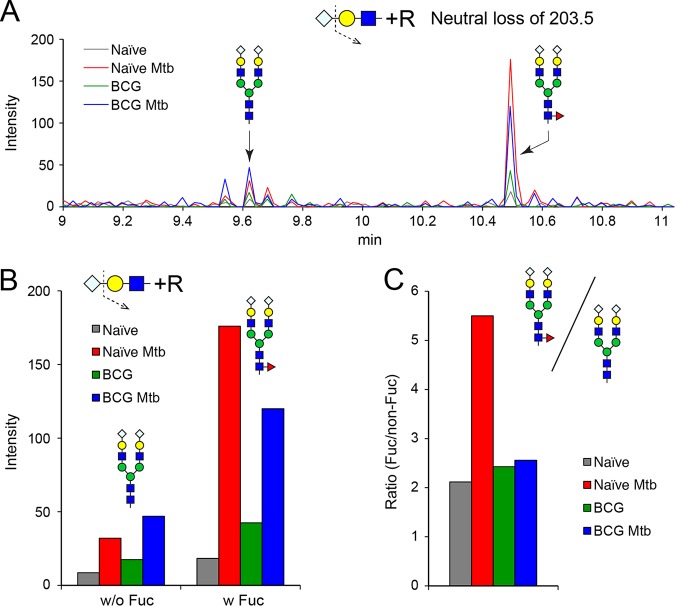
N-glycan composition of serum IgM. The IgM component was enriched and purified by immunoaffinity precipitation and SDS-PAGE from the indicated serum samples. Glycans were released by PNGase F digestion from the excised gel bands and analyzed by total ion monitoring (TIM) following permethylation. (A) TIM scans were filtered for loss of a NeuGc residue (Δ*m/z* = 203.5, doubly charged) to reveal the presence of the major sialylated biantennary glycans. The integrated signals associated with MS/MS fragments for the indicated glycan structures are shown as traces across scan time. (B) Signal intensities detected for the neutral loss fragment ions at *m/z* = 1,235.1 (doubly charged NeuGc_1_Gal_2_GlcNAc_2_Man_3_GlcNAc_2_) and at *m/z* 1,332.1 (doubly charged NeuGc_1_Gal_2_GlcNAc_2_ Man_3_GlcNAc_2_Fuc_1_) are shown for the indicated serum samples. (C) The ratio of the signal intensities for the fucosylated biantennary disialylated glycan was normalized to its nonfucosylated form for each of the serum samples to quantify the relative abundance changes. Black, red, green, and blue traces and bars indicate naive control, naive following *M. tuberculosis* infection, BCG control, and BCG following *M. tuberculosis* infection, respectively.

We purified IgM from serum in order to characterize the specific glycosylation changes on IgM glycopeptides in response to infection and immunization (Fig. 5A). Following in-gel tryptic digestion and subsequent glycan release by peptide-*N*-glycosidase F (PNGase F) digestion, we quantified the relative abundance of the 2 major N-glycans that we previously detected in serum (structures 11 and 17, Fig. 2). We note, for comparison, that two glycans dominate the characterized glycans found on human IgM; for both mouse and human IgM, these glycans are sialylated, biantennary complex structures, although reported to be undersialylated in human IgM (20). For each of the mouse IgM preparations we analyzed, the core fucosylated form of the most abundant biantennary complex glycan was more prevalent than the nonfucosylated form, regardless of infection or immunization status (Fig. 5B). Infection of naive mice increased the abundance of serum IgM by 1.5-fold (data not shown), while core fucosylation was increased more than 5-fold (Fig. 5C), indicating a significant shift in the processing of IgM glycans as a result of *M. tuberculosis* infection. However, BCG immunization blunted the increase in glycan fucosylation in response to *M. tuberculosis* infection by more than 50% (5.5-fold versus 2.5-fold, Fig. 5C).

## DISCUSSION

Most of the currently available tests to screen for TB are based on the detection of bacterially derived molecules. Therefore, these tests are not able to assess disease status based on host response (21). A largely unexplored area of investigation is whether glycomic or glycoproteomic alterations can be detected in host serum as a result of infection and whether these changes are informative regarding disease status (22). Recent work on human serum samples from TB-infected patients showed that immunoglobulin G, a major serum glycoprotein, is subject to differential glycosylation depending on disease status (4). We undertook a broader investigation of serum glycosylation, without bias for any particular protein, in mice infected with *M. tuberculosis*. We show that serum glycoproteins from animals infected with *M. tuberculosis* were altered in abundance and in the nature of their glycosylation.

We observed that protein fucosylation and sialylation are particularly sensitive to *M. tuberculosis* infection. We focused on ITIH4 and IgM proteins since lectin blotting and LC-MS/MS proteomic analysis indicated that they were impacted by infection (Fig. 4). Enhanced sialylation of the ITIH4 protein was detected in samples from *M. tuberculosis*-infected mice relative to naive ones. However, variations in ITIH4 abundance have also been associated with noninfectious inflammatory conditions such as hepatocellular carcinoma (18) or ischemic stroke (19), as well as with other infectious responses (17), suggesting that changes in the abundance or glycosylation of this serum protein are not specific for TB infection. We investigated the glycosylation of IgM at higher resolution by immunoaffinity purifying it from serum and then separating it from other contaminants by SDS-PAGE (Fig. 5). The glycans released from in-gel-digested IgM peptides were representative of the whole-serum glycan profile in that the two major glycans of IgM were also detected as the two major glycans of serum (structures 11 and 17 in Fig. 2). However, compared to the glycans of whole serum, the relative abundance of these two dominant glycans was reversed on IgM. The core-fucosylated glycan (structure 17) was detected at between 2- and 5.5-fold higher abundance than the nonfucosylated glycan (structure 11), depending on disease status. Furthermore, immunization with BCG modulates the magnitude of the change in fucosylation resulting from infection. Since antibody glycosylation can be greatly affected by immunization, inflammation, and disease progression (23), we cannot separate changes in glycosylation resulting from BCG vaccination from those resulting from differences in inflammation in vaccinated mice. Furthermore, it is possible that an analysis focused on the glycosylation of antibodies specific to mycobacterial antigens would reveal new and different patterns from those apparent from total immunoglobulin preparations. Nevertheless, we have observed that the glycosylation status of an individual serum protein may trend differently than would be predicted by the glycan profile of whole serum; combinations of seemingly contradictory elements (decreased fucosylated serum glycans but increased IgM protein fucosylation) may provide informative features for assessing disease status. In addition, we observed that one mouse from the group of BCG-vaccinated and *M. tuberculosis*-infected mice clustered with the vaccinated, uninfected group (Fig. 3). Tracing the identity of this mouse, we detected that it carried a higher number of CFU, suggesting that the context of glycosylation can also be microbe related.

The key result in this study was the demonstration that *M. tuberculosis* infection influenced host protein glycosylation in mice. The mechanism by which infection or vaccination modulates the structural characteristics of the glycome in a protein-specific manner is not currently understood. Altered Golgi trafficking, substrate availability, or enzyme targeting within the secretory apparatus is a possible point of regulation that could be affected by infection. Additionally, glycoprotein modifications could be edited or influenced by secreted hydrolases or glycosyltransferases in the serum (24, 25). Investigation of the regulation of such modifications by *M. tuberculosis* opens a new field that could lead to the discovery of novel biomarkers for the disease. To that end, the data presented here provide a baseline appreciation of the glycomic and glycoproteomic complexity associated with *M. tuberculosis* infection and disease status that can be expanded to include other glycoprotein targets and other species-specific features, including those that may be key for assessing human disease status. Determining the diagnostic and prognostic utility of quantitative and qualitative aspects IgM glycosylation in human *M. tuberculosis* infection will require correlation of these parameters with diseased states in clinical settings. Validation of such studies in a set of human serum samples, reflecting a range of different clinical scenarios, is under way. The outcome of such experiments will inform about the connection between mouse and human serology and the clinical relevance of these results.

## MATERIALS AND METHODS

### Strains.

M. bovis BCG Pasteur and *M. tuberculosis* H37Rv were grown in Middlebrook 7H9 supplemented with 10% (vol/vol) OADC enrichment (Becton, Dickinson Microbiology Systems, Sparks, MD), 0.5% (vol/vol) glycerol, with or without 0.05% (vol/vol) Tyloxapol for 14 days in a 5% CO_2_ incubator at 37°C.

### Immunization.

C57BL/6 female mice between 6 and 8 weeks old were purchased from Jackson Laboratories (Bar Harbor, ME). All procedures involving mice were reviewed and approved by the Animal Use and Care Committee of the Albert Einstein College of Medicine. Animals were maintained in a specific-pathogen-free animal facility under animal biosafety level 2 conditions for all experiments except for those involving infection with virulent *M. tuberculosis*, for which animal biosafety level 3 conditions were used. Animals were immunized subcutaneously with 1 million BCG bacteria 6 weeks prior to an infection with virulent *M. tuberculosis*. BCG was prepared for immunization as previously described (26).

### Mouse infection.

Aerogenic challenge was done using a whole-body exposure aerosol chamber (Mechanical Engineering Workshop) custom fitted to a class III biosafety cabinet (Baker) to deliver approximately 100 CFU per animal of *M. tuberculosis* (H37Rv). Immunized mice were infected 3 weeks after immunization. Mice were euthanized 30 days after challenge. Lungs of individual mice were aseptically removed and homogenized separately in 5 ml normal saline plus 0.05% Tyloxapol using a Seward Stomacher 80 blender (Tekmar). The homogenates were diluted serially and plated on Middlebrook 7H11 agar to determine CFU of *M. tuberculosis*. Dilutions of 10^−2^ and 10^−3^ and of 10^−2^ and 10^−1^ were plated when counting CFU in lungs and spleens, respectively. Animals infected with *M. tuberculosis* H37Rv were observed at least twice daily until they died or became moribund and were euthanized.

### Serum collection.

Blood was collected by cardiac puncture and centrifuged at 5,000 × *g*, and serum was stored at −80°C.

### Histology.

Lungs were removed and fixed in 10% neutral buffered formalin (Fisher Scientific, Fair Lawn, NJ). Tissues were embedded with paraffin, sectioned at 5-μm thickness, and stained with hematoxylin and eosin. Five different lung sections per mouse were analyzed. Slides were scanned with a Perkin-Elmer P250 high-capacity slide scanner (Waltham, MA) at 2,000 dots per inch (dpi). Digitized images were then analyzed using ImageJ software to calculate the total disease area occupied by granuloma and the percentage of lung surface affected by pneumonia as well as the number of infiltrates per lung. The total disease area for the entire lung section was measured by adding the values for each lesion. The total percentage of diseased tissue was calculated by dividing the total disease area by the entire lung section and multiplying by 100, using ImageJ software.

### SDS-PAGE and lectin immunoblotting.

The protein in 3 μl of mouse serum was precipitated with ice-cold 75% acetone, and the pellet was solubilized in 100 μl of Laemmli sample buffer. Five microliters of solubilized sample (equivalent to 0.15 μl of serum) was separated on a 4 to 15% TGX gradient gel (Bio-Rad), transferred to a polyvinylidene difluoride (PVDF) membrane, and then probed with biotinylated lectins (Sambucus nigra [SNA], Phaseolus vulgaris erythroagglutinin [E-PHA], Phaseolus vulgaris agglutinin [L-PHA], and Aleuria aurantia [AAL] lectins [Vector Laboratories, Burlingame, CA]) after blocking with 3% BSA in TBS. Probed membranes were incubated with Vectastain ABC-AP (Vector Laboratories), and bound lectin was detected with BCIP/NBT (Kirkegaard & Perry Laboratories, Inc., Gaithersburg, MD). Alternatively, PVDF membranes were incubated with anti-inter-alpha-trypsin inhibitor heavy chain H4 (ITIH4) antibody (ab180139; Abcam, Cambridge, MA), anti-mouse IgM conjugated to horseradish peroxidase (HRP) (A8786; Sigma), anti-ITIH4 antibody (sc-515353; Santa Cruz), goat anti-rabbit IgG conjugated to AP (Promega, Madison, WI), and rabbit anti-mouse IgG conjugated to AP (Promega, Madison, WI). Incubation with secondary antibodies was followed by detection with SigmaFast DAB with metal enhancer (Sigma, Saint Louis, MO) or incubation with the BCIP/NBT phosphatase substrate as appropriate.

### In-gel trypsin digestion and protein identification.

Identification of proteins of interest was performed as previously described (27, 28). Liquid chromatography-tandem mass spectrometry was performed with an LTQ-Orbitrap Discovery mass spectrometer (Thermo Fisher Scientific) equipped with a nanospray ionization source. The resulting data were searched against the mouse proteome database by using the Sequest algorithm (Proteome Discoverer 1.4; Thermo Scientific). Sequest parameters were set to allow 35.0 ppm of precursor ion mass tolerance and 0.8 Da of fragment ion tolerance with monoisotopic masses. Tryptic peptides were allowed with up to 2 missed internal cleavage sites, and differential modifications were allowed for carboxyamidomethylation of cysteine and oxidation of methionine. The resulting peptide data were filtered by charge versus cross-correlation (Xcorr) to result in a stringent false discovery rate of <1%.

### N-glycan analysis.

N-glycans released from serum glycoproteins by digestion with PNGase F (Prozyme, Hayward, CA) were permethylated prior to analysis by nanospray ionization multidimensional mass spectrometry (NSI-MS^n^) as previously described (29). Glycans released from equivalent amounts of protein were analyzed for comparison across treatments. Graphical representations of monosaccharide residues are presented in accordance with the broadly accepted Symbolic Nomenclature for Glycans (SNFG) (30), and glycan analysis was performed in accordance with the MIRAGE guidelines for glycomic studies (31).

### IgM immunoprecipitation and glycan analysis.

Aliquots of serum (50-μl total volume) were pooled from each sample type and diluted to 500 μl with TBS supplemented with 0.1% Triton X-100 and complete protease inhibitor mix (Roche). A suspension (300 μl) of anti-mouse Igμ chain antibody immobilized on agarose beads (A4540; Sigma) was added, and the mixture was incubated for 1 h at 4°C with agitation. Beads were subjected to sequential washes with 1.5 ml of PBS, and the bound IgMs were eluted with 1.5 ml of an 0.5 M acetic acid solution supplemented with 150 mM NaCl. After elution, salts were removed in a 10-kDa-cutoff spin column, and the resulting IgM preparations were resolved on a 4 to 15% SDS-PAGE gel and stained with Coomassie brilliant blue (CBB) G250. The IgM heavy chain band at approximately 70 kDa was excised and stored at 4°C until use. The gel band was minced into 1-mm cubes subjected to in-gel trypsin digestion. N-glycans were released from the resulting peptides by PNGase F digestion, permethylated, and analyzed by NSI-MS^n^ as described above. In addition to full MS analysis, permethylated glycans were also analyzed using the TIM (total ion monitoring) functionality of the Xcalibur instrument control and data acquisition software (Thermo Fisher). TIM analysis captures MS/MS data in discrete *m/z* windows across a user-defined mass range, providing validation of structural assignments associated with glycan peaks detected in full MS spectra. For each sample, TIM analysis generates 1,500 to 2,000 individual MS/MS spectra, which can be filtered for the presence of informative fragment ions. Filtered spectra were subjected to manual inspection and fragment ion annotation in order to validate the detected structural features.

### Statistics.

Standard one-way ANOVA followed by Tukey’s multiple-comparison test of the means was used to determine statistical significance of immune responses and protective efficacies of BCG vaccination and intensity of immunoblot bands. *P < *0.05 was considered statistically significant. Error bars represent SEM. Hierarchical clustering of glycan structural features was achieved as previously described (32).
